# First morphological characterization of ‘*Candidatus* Mycoplasma turicensis’ using electron microscopy

**DOI:** 10.1016/j.vetmic.2010.11.020

**Published:** 2011-05-05

**Authors:** Barbara Willi, Kristina Museux, Marilisa Novacco, Elisabeth M. Schraner, Peter Wild, Katrin Groebel, Urs Ziegler, Godelind A. Wolf-Jäckel, Yvonne Kessler, Catrina Geret, Séverine Tasker, Hans Lutz, Regina Hofmann-Lehmann

**Affiliations:** a Clinical Laboratory, University of Zurich, Zurich, Switzerland; b Clinic for Small Animal Internal Medicine, University of Zurich, Zurich, Switzerland; c Institute of Veterinary Anatomy, University of Zurich, Zurich, Switzerland; d Institute of Veterinary Bacteriology, Vetsuisse Faculty, University of Zurich, Zurich, Switzerland; e Centre for Microscopy and Image Analysis, University of Zurich, Zurich, Switzerland; f School of Clinical Veterinary Science, University of Bristol, Langford, Bristol, UK

**Keywords:** ‘*Candidatus* Mycoplasma turicensis’, ‘*Candidatus* Mycoplasma haemominutum’, Haemoplasma, Haemotropic *Mycoplasma*, Electron microscopy, Real-time PCR

## Abstract

At least three haemotropic mycoplasmas have been recognized in cats: *Mycoplasma haemofelis* (Mhf), ‘*Candidatus* Mycoplasma haemominutum’ (CMhm) and ‘*Candidatus* M. turicensis’ (CMt). The latter was originally identified in a Swiss pet cat with haemolytic anaemia and shown to be prevalent in domestic cats and wild felids worldwide using molecular methods. So far, there has been no confirmatory morphological evidence of the existence of CMt presumably due to low blood loads during infection while CMhm has only been characterized by light microscopy with discrepant results. This study aimed to provide for the first time electron microscopic characteristics of CMt and CMhm and to compare them to Mhf. Blood samples from cats experimentally infected with CMt, CMhm and Mhf were used to determine copy numbers in blood by real-time PCR and for transmission and scanning electron microscopy. High resolution scanning electron microscopy revealed CMt and CMhm to be discoid-shaped organisms of 0.3 μm in diameter attached to red blood cells (RBCs). In transmission electron microscopy of CMt, an oval organism of about 0.25 μm with several intracellular electron dense structures was identified close to the surface of a RBC. CMhm and CMt exhibited similar morphology to Mhf but had a smaller diameter. This is the first study to provide morphological evidence of CMt thereby confirming its status as a distinct haemoplasma species, and to present electron microscopic features of CMhm.

## 1 Introduction

Haemotropic mycoplasmas (aka haemoplasmas) are uncultivable, cell wall-free bacteria that attach to the red blood cells (RBCs) of mammalian hosts and can induce severe haemolytic anaemia in infected animals (Messick, 2003, 2004). Two feline haemoplasmas were originally recognized: *Mycoplasma haemofelis* (Mhf) and ‘*Candidatus* Mycoplasma haemominutum’ (CMhm) (Berent et al., 1998; Foley et al., 1998; Messick et al., 1998; Tasker et al., 2001). Some years ago, a third feline haemoplasma, ‘*Candidatus* Mycoplasma turicensis’ (CMt), was discovered in a Swiss pet cat with haemolytic anaemia (Willi et al., 2005). Recent studies have documented CMt infections in pet cats and wild felids worldwide using molecular methods (Fujihara et al., 2007; Gentilini et al., 2009; Kamrani et al., 2008; Peters et al., 2008; Sykes et al., 2007; Willi et al., 2006a,b, 2007). However, there has been no confirmed morphological evidence of the existence of CMt, likely due to the low blood loads seen during infection (Museux et al., 2009; Peters et al., 2008; Tasker et al., 2009; Willi et al., 2006a,b). In contrast, Mhf and CMhm have been successfully identified by light microscopy. However, discrepant results have been published concerning the morphological features of CMhm and no morphological characteristics of CMhm based on electron microscopy are yet available.

The goal of this study was to provide for the first time electron microscopic features of CMt and CMhm by means of scanning electron microscopy (SEM) and transmission electron microscopy (TEM) on blood samples from experimentally infected cats. The morphologic characteristics of CMt and CMhm were compared to those of Mhf.

## 2 Materials and methods

### 2.1 Experimental infection and PCR analysis

The experimental infections of the cats were performed for unrelated studies. Briefly, two SPF cats (Cats Z and 1, Table 1) were experimentally infected intraperitoneally or subcutaneously with CMt as previously described (Museux et al., 2009). For experimental CMhm infection, one SPF cat (Cat Q1, Table 1) was inoculated intraperitoneally with 1 ml of EDTA blood in 20% dimethyl sulfoxide (DMSO, Sigma–Aldrich, Buchs, Switzerland) as described (Geret et al., submitted for publication). The latter blood was positive for CMhm (6.9 × 10^5^ copies/ml) and feline leukaemia virus (FeLV, 9.8 × 10^7^ copies virus/ml). For experimental Mhf infection, one SPF cat (Cat QLA5, Table 1) was inoculated intraperitoneally with 2 ml of Mhf-PCR positive blood (10^9^ copies/ml, preserved in 20% DMSO, Wolf-Jäckel et al., 2010) from the experimentally infected cat HF3 (Tasker et al., 2009). The cats were kept in groups with other cats either uninfected or infected with the same infectious agents in a confined university facility under etiologically and hygienically ideal conditions as described (Museux et al., 2009). The experiments were performed according to the law and officially approved by the veterinary office of the canton Zurich (TVB 101/2007). Prior to inoculation, blood and serum samples, and conjunctival, oropharyngeal and rectal swabs were collected to verify the SPF status of the cats as previously described (Museux et al., 2009). After experimental infection, the cats were monitored for blood loads of the inoculated haemoplasma species: total nucleic acid (TNA) was extracted from 100 μl of EDTA-anticoagulated blood, analysed by specific TaqMan real-time PCR assays and absolute quantification performed using plasmid standards as described previously (Willi et al., 2006a, 2005). In addition, TNA from blood collected from the cats after successful haemoplasma infection was tested with feline haemoplasma-specific TaqMan PCR assays as described to confirm absence of haemoplasma co-infections (Willi et al., 2006a, 2005).

### 2.2 Haematology and blood smear preparation

Haemograms from EDTA-anticoagulated blood were performed regularly using a Cell-Dyn 3500 system (Abbott, Baar, Switzerland) or a Sysmex XT-2000iV (Sysmex Corporation, Kobe, Japan, Weissenbacher et al., 2010). Blood smears were prepared from the CMt- and Mhf-infected cats (Cats Z, 1 and QLA5). For blood smear preparation, EDTA-anticoagulated blood was stained with Giemsa using an automated slide stainer or stained manually with Diff-Quick as reported previously (Museux et al., 2009). Additionally, smears were prepared from non-anticoagulated blood immediately after collection and Diff-Quick stained. All smears were evaluated by light microscopy for the presence of haemoplasmas.

### 2.3 Electron microscopy

Blood samples for electron microscopic studies were collected from Cats Z, 1, Q1 and QLA5 at the time or four days to two weeks after peak bacteraemia. Blood samples were anticoagulated with lithium-heparin or Alsever's solution; anticoagulation with Alsever's solution was performed using a 1:1 mixture of whole blood with the anticoagulant. Anticoagulated blood samples were fixed within 10 min of collection. For SEM, a total of 600 μl of anticoagulated blood was added to 2.5 ml of 0.1 M Na/K-phosphate, pH 7.4, and the mixture slowly added to 5 ml of 2.5% glutaraldehyde in 0.1 M Na/K-phosphate. The homogeneous mixture was centrifuged at 1400 × *g* for 10 min and the supernatant was discarded. The resulting pellet was resuspended in 2.5 ml of 0.1 M Na/K-phosphate and stored at 4 °C. For final preparation, cells were centrifuged at 600 × *g* for 5 min, resuspended in 1% osmium tetroxide in phosphate buffered saline (PBS), and washed once with PBS by centrifugation at 600 g for 5 min. A total of 100 μl of resuspended cells in PBS were centrifuged in a Shandon Cytospin 2 (DAKO, Baar, Switzerland) onto carbon coated cover slips at 300 g for 8 min, dehydrated in a graded acetone series, critical point dried (Baltec CPD 030, Leica Microsystems, Heerbrugg, Switzerland), sputter coated with 5 nm gold (Baltec SCD 010), and imaged in a field emission scanning electron microscope (Leo Gemini 50 VP, Zeiss, Oberkochen, Germany) at an acceleration voltage of 5 kV. For TEM, a total of 120 μl of anticoagulated blood was added to 1 ml of 0.1 M Na/K-phosphate, pH 7.4, and the mixture was slowly added to 1 ml of 2.5% glutaraldehyde in 0.1 M Na/K-phosphate. The homogeneous mixture was centrifuged at 3600 × *g* for 25 min, the supernatant removed, and the pellet stored in 0.1 M Na/K-phosphate at 4 °C until final preparation. Cell pellets were post-fixed with 1% osmium tetroxide in 0.1 M Na/K-phosphate, dehydrated in a graded ethanol series, transferred to acetone for embedding in epon, and polymerized at 60 °C for 2.5 days. Ultrathin sections were stained with uranyl acetate and lead citrate and examined at an acceleration voltage of 100 kV in a Philips CM 12 transmission electron microscope (Philips, Eindhoven, the Netherlands) equipped with a low scan CCD camera (Gatan, Pleasanton, CA, USA).

## 3 Results

### 3.1 Experimental infection

Experimental infection of Cats Z, 1, Q1 and QLA5 was successful (Geret et al., submitted for publication; Museux et al., 2009; Wolf-Jäckel et al., 2010). Peak haemoplasma copy numbers were lower in the two CMt-infected Cats Z and 1 (5.77 × 10^6^ copies/ml of blood on day 15 p.i. and 9.04 × 10^5^ copies/ml of blood on day 23 p.i., respectively) than in the Mhf-infected Cat QLA5 (4.42 × 10^8^ copies/ml of blood on day 10 p.i.) and the CMhm-infected Cat Q1 (1.35 × 10^8^ copies/ml of blood at 6 weeks p.i.). All four cats were free of haemoplasma co-infections as determined by TaqMan real-time PCR assays.

### 3.2 Light microscopic evaluation of blood smears for CMt

None of the blood smears from the two CMt-infected cats, independent of the preparation method used, showed structures compatible with haemoplasma organisms. For comparison, blood smears from the Mhf-infected Cat QLA5 prepared on days 10 and 11 p.i. showed structures consistent with haemoplasma organisms (data not shown).

### 3.3 SEM characteristics of CMt, CMhm and Mhf

**Fig. 1:**
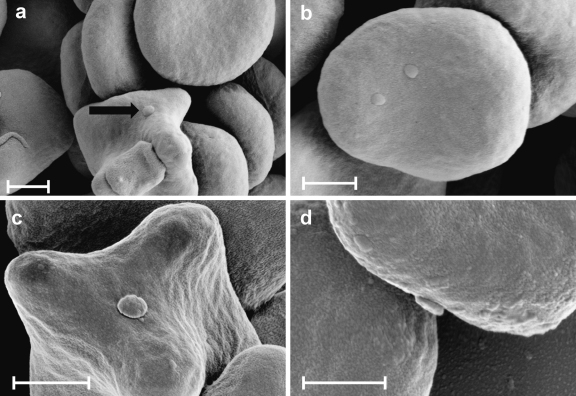
(a–d) SEM images of RBCs from blood anticoagulated with Alsever's solution collected from Cat 1 on day 23 after experimental infection with CMt. Discoid-shaped organisms, about 0.3 μm in diameter, are attached to the surface of RBCs (indicated by an arrow in a). Bars represent 1 μm.

**Fig. 2:**
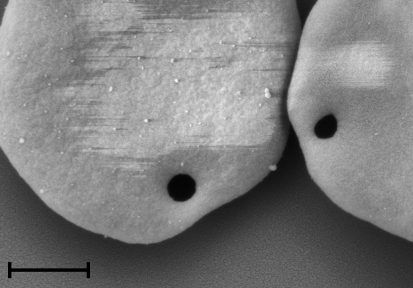
SEM image of RBCs from lithium-heparin-anticoagulated blood collected from Cat Z 19 days after experimental infection with CMt. Circular contrast-free regions of about 0.3 μm on two RBCs are visible. Bar represents 1 μm.

**Fig. 3:**
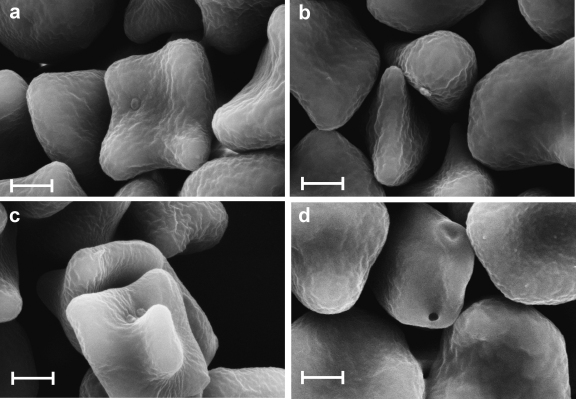
(a–d) SEM images of RBCs from blood anticoagulated with Alsever's solution collected from Cat Q1 on day 56 after experimental infection with CMhm. Discoid-shaped organisms, about 0.3 μm in diameter, are attached to the surface of RBCs. A circular contrast-free region of about 0.3 μm in diameter is seen on the surface of a RBC in Fig. 4 d. Bars represent 1 μm.

**Fig. 4:**
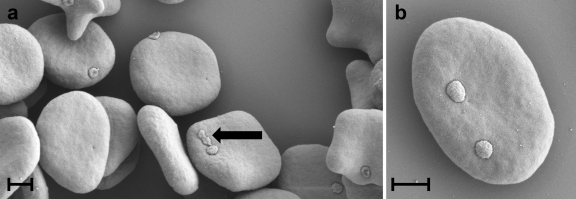
(a, b) SEM images of RBCs from blood anticoagulated with Alsever's solution collected from Cat QLA5 10 days after experimental infection with Mhf. Several discoid-shaped organisms of about 0.5 μm in diameter are attached to RBCs. An organism in binary fission is indicated by an arrow. Bars represent 1 μm.

High resolution SEM revealed structures compatible with haemoplasmas epicellularly attached to RBCs in the blood sample collected from the CMt-infected Cat 1 (Fig. 1 a-d). These discoid-shaped structures were about 0.3 μm in diameter. In the CMt-infected Cat Z, contrast-free, not gold-coated, circular regions of about 0.3 μm in diameter were identified on the surface of several RBCs (Fig. 2). In the CMhm-infected Cat Q1, discoid-shaped structures of about 0.3 μm in diameter were identified on several RBCs (Fig. 3a–c). Some RBCs showed also contrast-free, not gold-coated, circular regions of about 0.3 μm on their surface (Fig. 3d). In the Mhf-infected Cat QLA5, SEM revealed discoid-shaped organisms 0.5 μm in diameter on the surface of RBCs (Fig. 4a and b). Some of these structures showed binary fission (Fig. 4 a, indicated by an arrow).

### 3.4 TEM characteristics of CMt and Mhf

**Fig. 5:**
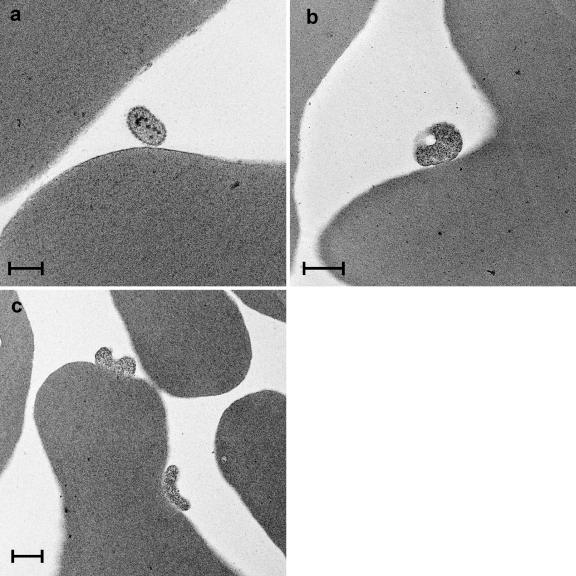
TEM images. (a) Lithium-heparin-anticoagulated blood collected from Cat Z on day 19 after experimental infection with CMt. An oval structure of about 0.25 μm in length with intracellular round electron dense particles is located close to the surface of a RBC. Bar represents 0.2 μm. (b, c) Blood sample anticoagulated with Alsever's solution and collected from Cat QLA5 10 days after experimental infection with Mhf. Structures of about 0.5–0.6 μm in diameter are tightly attached to the surface of RBCs. Bars represent 0.5 μm.

An oval structure of about 0.25 μm was identified by TEM close to the surface of a RBC of Cat Z experimentally infected with CMt (Fig. 5 a). The latter structure showed several intracellular electron dense round particles. In the Mhf-infected Cat QLA5, structures of about 0.5–0.6 μm in diameter were found closely attached to the surface of RBCs (Fig. 5b and c). While the latter structures most closely resemble haemoplasmas, the one in Fig. 5a might also represent a section through a protrusion of a blood platelet.

## 4 Discussion

The present study provides for the first time morphological evidence of CMt. So far, the organism has only been detected using sensitive molecular methods. By using electron microscopy, the morphology of the organisms found attached to the RBCs of Cat 1 experimentally infected with CMt was similar to that reported for Mhf as well as for other haemoplasma species (Demaree and Nessmith, 1972; Groebel et al., 2009; Jain and Keeton, 1973; Maede and Sonoda, 1975; McKee et al., 1973; Neimark and Kocan, 1997). This suggests that the herein described structures indeed represent CMt. In SEM, CMt seems to exhibit a slightly smaller diameter (0.3 μm) than previously and herein reported for Mhf (0.5 μm) (Jain and Keeton, 1973; Maede and Sonoda, 1975).

SEM revealed that CMhm exhibits a similar morphology and size as CMt (0.3 μm). A similar size for CMhm has been reported by an earlier study based on light microscopy (Foley and Pedersen, 2001), whereas another report determined a larger size of approximately 0.6 μm for one ‘*Candidatus* M. haemominutum’ strain (Tasker et al., 2001). However, size determination can be hampered by methodological inaccuracies.

SEM of blood samples from CMt- and CMhm-infected cats revealed contrast-free, not gold-coated circular regions, about 0.3 μm in diameter, on the surface of several RBCs. Previous studies have also reported ‘pock-marks’ or ‘holes’ on the surface of RBCs in haemoplasma-infected animals (Jain and Keeton, 1973; Yang et al., 2007). Some authors have hypothesized that these lesions are due to the detachment of haemoplasmas from the RBC surface, and that this could be responsible for the increased osmotic fragility of RBCs commonly observed during haemoplasma infections (Maede, 1975; Yang et al., 2007). Indeed, an increase in RBC osmotic fragility has also been reported in cats experimentally infected with CMt (Willi et al., 2005). In the present study, detachment of organisms may also have been initiated during preparation, e.g. during osmium tetroxide fixation depending on the buffer used prior to osmium fixation (Hayat, 2000). Detachment of haemoplasmas has also been reported with EDTA as an anticoagulant (Alleman et al., 1999).

In agreement with previous studies, we were unable to identify CMt in blood smears by light microscopy, independent of the blood smear preparation method used. We believe this to be due to the low CMt blood loads seen during infection: the maximal blood loads of 5.77 × 10^6^ copies/ml of blood in Cat Z corresponded to only one CMt copy per 10^3^ to 10^4^ RBCs (Museux et al., 2009), whereas at peak bacteraemia in Cat QLA5, one could expect one Mhf copy per 10–100 RBCs (Table 1). Therefore, light microscopic evaluation of blood smears is a very insensitive method to diagnose CMt infections and PCR represents the diagnostic method of choice to detect infections with this agent.

## 5 Conclusion

We conclude from the present study that CMt and CMhm show morphological characteristics similar to Mhf but they are of smaller size. Given the typically low CMt blood loads during infection, light microscopy of blood smears has a very low diagnostic sensitivity and PCR represents the method of choice to diagnose CMt infections.

## Figures and Tables

**Table 1:** Experimental infection of Cats Z, 1, Q1 and QLA5 and characteristics of samples collected for electron microscopy.

Cat	Agent^a^	Route of infection^b^	Age at infection (months)	Characteristics of samples collected for electron microscopy				
				Day of blood collection^c^	Haemoplasma blood load^d^	PCV (%)	RBC count^e^	Anticoagulant
Z	CMt	i.p.	37	19	4.38 × 10^6^	24	5.68 × 10^6^	Lithium-heparin
1	CMt	s.c.	12	23	9.04 × 10^5^	28	6.55 × 10^6^	Alsever's solution
Q1	CMhm	i.p.	3	56	5.65 × 10^6^	28	6.91 × 10^6^	Alsever's solution
QLA5	Mhf	i.p.	33	10	4.42 × 10^8^	24	5.59 × 10^6^	Alsever's solution

a CMt: ‘*Candidatus* M. turicensis’, CMhm: ‘*Candidatus* M. haemominutum’, Mhf: *M. haemofelis*.

b i.p.: intraperitoneally, s.c.: subcutaneously.

c Day post-infection.

d Copies/ml of blood.

e Cells/μl of blood.

## References

[bib0005] Alleman A.R., Pate M.G., Harvey J.W., Gaskin J.M., Barbet A.F. (1999). Western immunoblot analysis of the antigens of *Haemobartonella felis* with sera from experimentally infected cats. J. Clin. Microbiol..

[bib0010] Berent L.M., Messick J.B., Cooper S.K. (1998). Detection of *Haemobartonella felis* in cats with experimentally induced acute and chronic infections, using a polymerase chain reaction assay. Am. J. Vet. Res..

[bib0015] Demaree R.S., Nessmith W.B. (1972). Ultrastructure of *Haemobartonella felis* from a naturally infected cat. Am. J. Vet. Res..

[bib0020] Foley J.E., Harrus S., Poland A., Chomel B., Pedersen N.C. (1998). Molecular, clinical, and pathologic comparison of two distinct strains of *Haemobartonella felis* in domestic cats. Am. J. Vet. Res..

[bib0025] Foley J.E., Pedersen N.C. (2001). ’*Candidatus* Mycoplasma haemominutum’, a low-virulence epierythrocytic parasite of cats. Int. J. Syst. Evol. Microbiol..

[bib0030] Fujihara M., Watanabe M., Yamada T., Harasawa R. (2007). Occurrence of ‘*Candidatus* Mycoplasma turicensis’ infection in domestic cats in Japan. J. Vet. Med. Sci..

[bib0035] Gentilini F., Novacco M., Turba M.E., Willi B., Bacci M.L., Hofmann-Lehmann R. (2009). Use of combined conventional and real-time PCR to determine the epidemiology of feline haemoplasma infections in northern Italy. J. Feline Med. Surg..

[bib0040] Geret, C.P., Cattori, V., Meli, M.L., Riond, B., Martínez, F., López, G., Vargas, A., Simón, M.A., López-Bao, J.V., Hofmann-Lehmann, R., Lutz, H., submitted for publication. Feline leukemia virus outbreak in the critically endangered Iberian lynx (Lynx pardinus): molecular characterization of the virus and experimental transmission. 10.1007/s00705-011-0925-z 21302124

[bib0045] Groebel K., Hoelzle K., Wittenbrink M.M., Ziegler U., Hoelzle L.E. (2009). Mycoplasma suis invades porcine erythrocytes. Infect. Immun..

[bib0050] Hayat M.A. (2000). Principles and Techniques of Electron Microscopy.

[bib0055] Jain N.C., Keeton K.S. (1973). Scanning electron microscopic features of *Haemobartonella felis*. Am. J. Vet. Res..

[bib0060] Kamrani A., Parreira V.R., Greenwood J., Prescott J.F. (2008). The prevalence of *Bartonella*, hemoplasma, and *Rickettsia felis* infections in domestic cats and in cat fleas in Ontario. Can. J. Vet. Res..

[bib0065] Maede Y. (1975). Studies on feline haemobartonellosis. IV. Lifespan of erythrocytes of cats infected with *Haemobartonella felis*. Nippon Juigaku Zasshi.

[bib0070] Maede Y., Sonoda M. (1975). Studies on feline haemobartonellosis. III. Scanning electron microscopy of *Haemobartonella felis*. Nippon Juigaku Zasshi.

[bib0075] McKee A.E., Ziegler R.F., Giles R.C. (1973). Scanning and transmission electron microscopy of *Haemobartonella canis* and *Eperythrozoon ovis*. Am. J. Vet. Res..

[bib0080] Messick J.B. (2003). New perspectives about Hemotrophic mycoplasma (formerly, *Haemobartonella* and *Eperythrozoon* species) infections in dogs and cats. Vet. Clin. North Am. Small Anim. Pract..

[bib0085] Messick J.B. (2004). Hemotrophic mycoplasmas (hemoplasmas): a review and new insights into pathogenic potential. Vet. Clin. Pathol..

[bib0090] Messick J.B., Berent L.M., Cooper S.K. (1998). Development and evaluation of a PCR-based assay for detection of *Haemobartonella felis* in cats and differentiation of *H. felis* from related bacteria by restriction fragment length polymorphism analysis. J. Clin. Microbiol..

[bib0095] Museux K., Boretti F.S., Willi B., Riond B., Hoelzle K., Hoelzle L.E., Wittenbrink M.M., Tasker S., Wengi N., Reusch C.E., Lutz H., Hofmann-Lehmann R. (2009). *In vivo* transmission studies of ‘*Candidatus* Mycoplasma turicensis’ in the domestic cat. Vet. Res..

[bib0100] Neimark H., Kocan K.M. (1997). The cell wall-less rickettsia *Eperythrozoon wenyonii* is a *Mycoplasma*. FEMS Microbiol. Lett..

[bib0105] Peters I.R., Helps C.R., Willi B., Hofmann-Lehmann R., Tasker S. (2008). The prevalence of three species of feline haemoplasmas in samples submitted to a diagnostics service as determined by three novel real-time duplex PCR assays. Vet. Microbiol..

[bib0110] Sykes J.E., Drazenovich N.L., Ball L.M., Leutenegger C.M. (2007). Use of conventional and real-time polymerase chain reaction to determine the epidemiology of hemoplasma infections in anemic and nonanemic cats. J. Vet. Intern. Med..

[bib0115] Tasker S., Helps C.R., Belford C.J., Birtles R.J., Day M.J., Sparkes A.H., Gruffydd-Jones T.J., Harbour D.A. (2001). 16S rDNA comparison demonstrates near identity between an United Kingdom *Haemobartonella felis* strain and the American California strain. Vet. Microbiol..

[bib0120] Tasker S., Peters I.R., Papasouliotis K., Cue S.M., Willi B., Hofmann-Lehmann R., Gruffydd-Jones T.J., Knowles T.G., Day M.J., Helps C.R. (2009). Description of outcomes of experimental infection with feline haemoplasmas: copy numbers, haematology, Coombs’ testing and blood glucose concentrations. Vet. Microbiol..

[bib0125] Weissenbacher S., Riond B., Hofmann-Lehmann R., Lutz H. (2010). Evaluation of a novel haematology analyser for use with feline blood. Vet. J..

[bib0130] Willi B., Boretti F.S., Baumgartner C., Tasker S., Wenger B., Cattori V., Meli M.L., Reusch C.E., Lutz H., Hofmann-Lehmann R. (2006). Prevalence, risk factor analysis, and follow-up of infections caused by three feline hemoplasma species in cats in Switzerland. J. Clin. Microbiol..

[bib0135] Willi B., Boretti F.S., Cattori V., Tasker S., Meli M.L., Reusch C., Lutz H., Hofmann-Lehmann R. (2005). Identification, molecular characterization, and experimental transmission of a new hemoplasma isolate from a cat with hemolytic anemia in Switzerland. J. Clin. Microbiol..

[bib0140] Willi B., Boretti F.S., Meli M.L., Bernasconi M.V., Casati S., Hegglin D., Puorger M., Neimark H., Cattori V., Wengi N., Reusch C.E., Lutz H., Hofmann-Lehmann R. (2007). Real-time PCR investigation of potential vectors, reservoirs, and shedding patterns of feline hemotropic mycoplasmas. Appl. Environ. Microbiol..

[bib0145] Willi B., Tasker S., Boretti F.S., Doherr M.G., Cattori V., Meli M.L., Lobetti R.G., Malik R., Reusch C.E., Lutz H., Hofmann-Lehmann R. (2006). Phylogenetic analysis of “*Candidatus* Mycoplasma turicensis” isolates from pet cats in the United Kingdom, Australia, and South Africa, with analysis of risk factors for infection. J. Clin. Microbiol..

[bib0150] Wolf-Jäckel G.A., Jäckel C., Museux K., Hoelzle K., Tasker S., Lutz H., Hofmann-Lehmann R. (2010). Identification, characterization, and application of a recombinant antigen for the serological diagnosis of feline hemotropic mycoplasma infections. Clin. Vac. Immunol..

[bib0155] Yang Z., Yuan C., Yu F., Hua X. (2007). Haemotrophic mycoplasma: review of aetiology and prevalence. Rev. Med. Microbiol..

